# A novel cuproptosis-related lncRNAs signature predicts prognosis in bladder cancer

**DOI:** 10.18632/aging.204861

**Published:** 2023-07-09

**Authors:** Lingfeng Wu, Wei Chen, Yifang Cao, Bin Chen, Yi He, Xueping Wang

**Affiliations:** 1 Department of Urology, The Affiliated Hospital of Jiaxing University, Jiaxing, Jiangzhe 314000, China

**Keywords:** cuproptosis, BLCA, lncRNAs, prognosis, drug therapy

## Abstract

This study constructed a novel cuproptosis-related lncRNAs signature to predict the prognosis of BLCA patients. The Cancer Genome Atlas (TCGA) database was used to retrieve the RNA-seq data together with the relevant clinical information. The cuproptosis-related genes were first discovered. The cuproptosis-related lncRNAs were then acquired by univariate, the least absolute shrinkage and selection operator (LASSO) and multivariate Cox regression analysis to create a predictive signature. An eight cuproptosis-related lncRNAs (AC005261.1, AC008074.2, AC021321.1, AL024508.2, AL354919.2, ARHGAP5-AS1, LINC01106, LINC02446) predictive signature was created. Compared with the low-risk group, the prognosis was poorer for the high-risk group. The signature served as an independent overall survival (OS) predictor. Receiver operating characteristic (ROC) curve indicated that the signature demonstrated superior predictive ability, as evidenced by the area under the curve (AUC) of 0.782 than the clinicopathological variables. When we performed a subgroup analysis of the different variables, the high-risk group’s OS for BLCA patients was lower than that of the low-risk group’s patients. Gene Set Enrichment Analysis (GSEA) showed that high-risk groups were clearly enriched in many immune-related biological processes and tumor-related signaling pathways. Single sample gene set enrichment analysis (ssGSEA) revealed that the immune infiltration level was different between the two groups. Finally, quantitative RT-PCR showed that AC005261.1, AC021321.1, AL024508.2, LINC02446 and LINC01106 were lowly expressed in tumor cells, while ARHGAP5-AS1 showed the opposite trend. In summary, the predictive signature can independently predict the prognosis and provide clinical treatment guidance for BLCA patients.

## INTRODUCTION

Bladder Urothelial Carcinoma (BLCA) is the tenth most prevalent malignant tumor in the world, accounting for about 573,000 new cases and 213,000 new fatalities in 2020 [1]. BLCA is typically split into muscle invasive bladder cancer and non-muscle invasive forms (MIBC and NMIBC, respectively) [2–4]. Because these two forms have completely different biological characteristics, there are significant differences in pathogenesis, overall survival and treatment options [5]. As is known to all, radical cystectomy is the main treatment strategy for patients with BLCA. However, it has a high incidence of distant metastases, a high postoperative recurrence rate and a low five-year survival rate [6]. Age has been found to be an independent risk factor for bladder cancer development. Various demographic studies have shown an overall 11-fold increase in cancer incidence and a 15-fold increase in cancer mortality for individuals aged 65 and over compared to those under 65 [7]. The average age of diagnosis for BLCA patients was 73, meaning it remains the most intensive and expensive cancer to treat in the elderly [8]. Therefore, there is an urgent need to identify new and useful biomarkers, as well as to create prognostic models with greater accuracy for early detection and therapy.

Copper is an indispensable trace element in biological processes, and its deficiency or excess may lead to diseases [9]. Recent studies have shown that compared with healthy controls, the levels of copper in the serum and tumor tissues of cancer patients are significantly increased [10–12]. Due to its double-edged sword function, copper is an essential enzymatic cofactor but also causes cell death. Therefore, it is expected to become a new therapeutic target by increasing the accumulation of intracellular copper to specifically kill cancer cells [11]. Recently, Tsvetkov et al. reported a novel copper-dependent cell death mode named “cuproptosis” in *Science* which was different from the known cell death mechanism (such as apoptosis, ferroptosis, necroptosis, autophagy, pyroptosis and so on) [13]. Copper can directly bind to lipid acylated components of the tricarboxylic acid (TCA) cycle to induce cuproptosis. This finally results in cell death due to proteotoxic stress [13].

LncRNAs, which are transcripts greater than 200 nucleotides that don’t code for proteins, are differentiated from small non-coding RNAs [14]. It has been reported that lncRNAs influence both the development and progression of tumors [15, 16]. LncRNAs have also been found to modulate tumor immune responses [17, 18]. In addition, another study found that lncRNAs can be used as prognostic markers for tumors [19]. Recently, some studies found that lncRNAs were related to cuproptosis in many tumors, such as lung adenocarcinoma, hepatocellular carcinoma, osteosarcoma, kidney renal papillary cell carcinoma, and so on [20–24]. Therefore, it is necessary to develop prognostic biomarkers for early diagnosis and treatment by establishing lncRNA models in BLCA.

In this work, we constructed and validated a predictive signature for evaluating the prognosis, tumor immune infiltration and drug response in BLCA patients based on cuproptosis-related lncRNAs. Our work may contribute to the early diagnosis and treatment of BLCA patients.

## RESULTS

**Figure 1:**
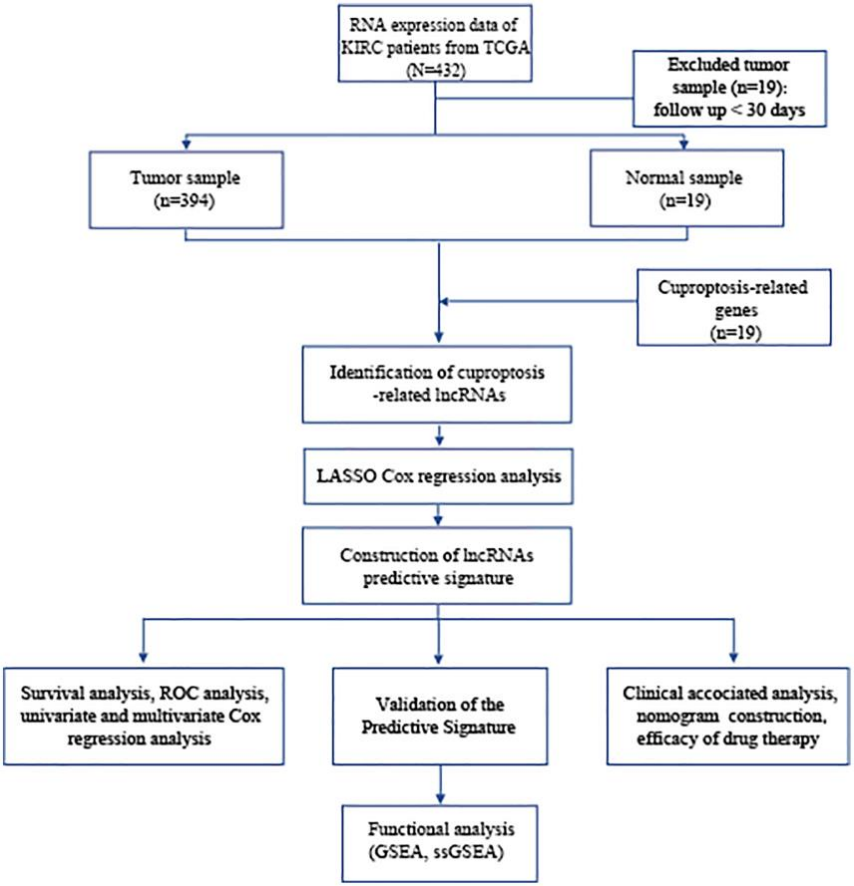
**Flowchart of data collection and analysis.** Abbreviations: BLCA: Bladder urothelial carcinoma; TCGA: The Cancer Genome Atlas; KEGG: Kyoto Encyclopedia of Genes and Genomes; GO: Gene Ontology; lncRNAs: long noncoding RNAs; LASSO: least absolute shrinkage and selection operator; ROC: receiver operating characteristic; GSEA: gene enrichment analysis; ssGSEA: single-sample gene set enrichment analysis.

The study’s flowchart is depicted in Figure 1, with a total of 394 BLCA patients enrolled from the TCGA-BLCA cohort, and their clinical characteristics outlined in detail in Table 1.

**Table 1:** The clinical characteristics of patients in different cohorts.

**Variables**	**TCGA cohort** **(*n* = 394)**	**Training cohort** **(*n* = 198)**	**Testing cohort** **(*n* = 196)**
Age (%)
≤65	158 (40.1%)	85 (42.9%)	73 (37.2%)
>65	236 (59.9%)	113 (57.1%)	123 (62.8%)
Gender (%)
Female	103 (26.1%)	49 (33.6%)	54 (27.6%)
Male	291 (73.9%)	149 (66.4%)	142 (72.4%)
Grade (%)
High Grade	373 (94.7%)	184 (92.9%)	189 (96.4%)
Low Grade	18 (4.6%)	12 (6.1%)	6 (3.1%)
Unknow	3 (0.7%)	2 (1%)	1 (0.5%)
Stage (%)
I + II	125 (31.7%)	66 (33.3%)	59 (30.1%)
III + IV	267 (67.8%)	130 (65.7%)	137 (69.9%)
Unknow	2 (0.5%)	2 (1%)	0 (0%)
T (%)
T0	1 (0.3%)	0 (0%)	1 (0.5%)
T1 + T2	115 (29.2%)	61 (30.8%)	54 (27.6%)
T3 + T4	246 (62.4%)	123 (62.1%)	123 (62.8%)
TX + Unknow	32 (8.1%)	14 (7.1%)	18 (9.1%)
*N* (%)
N0	228 (57.9%)	120 (60.6%)	108 (55.1%)
N1–3	125 (31.7%)	62 (31.3%)	63 (32.1%)
NX + Unknow	41 (10.4%)	16 (8.1%)	25 (12.8%)
M (%)
M0	188 (47.7%)	94 (47.5%)	94 (48.0%)
M1	10 (2.6%)	5 (2.5%)	5 (2.5%)
MX + Unknow	196 (49.7%)	99 (50%)	97 (49.5%)

Abbreviations: T: tumor; M: metastasis; N: lymph node.

### Construction of the cuproptosis-related lncRNA predictive signature

**Figure 2:**
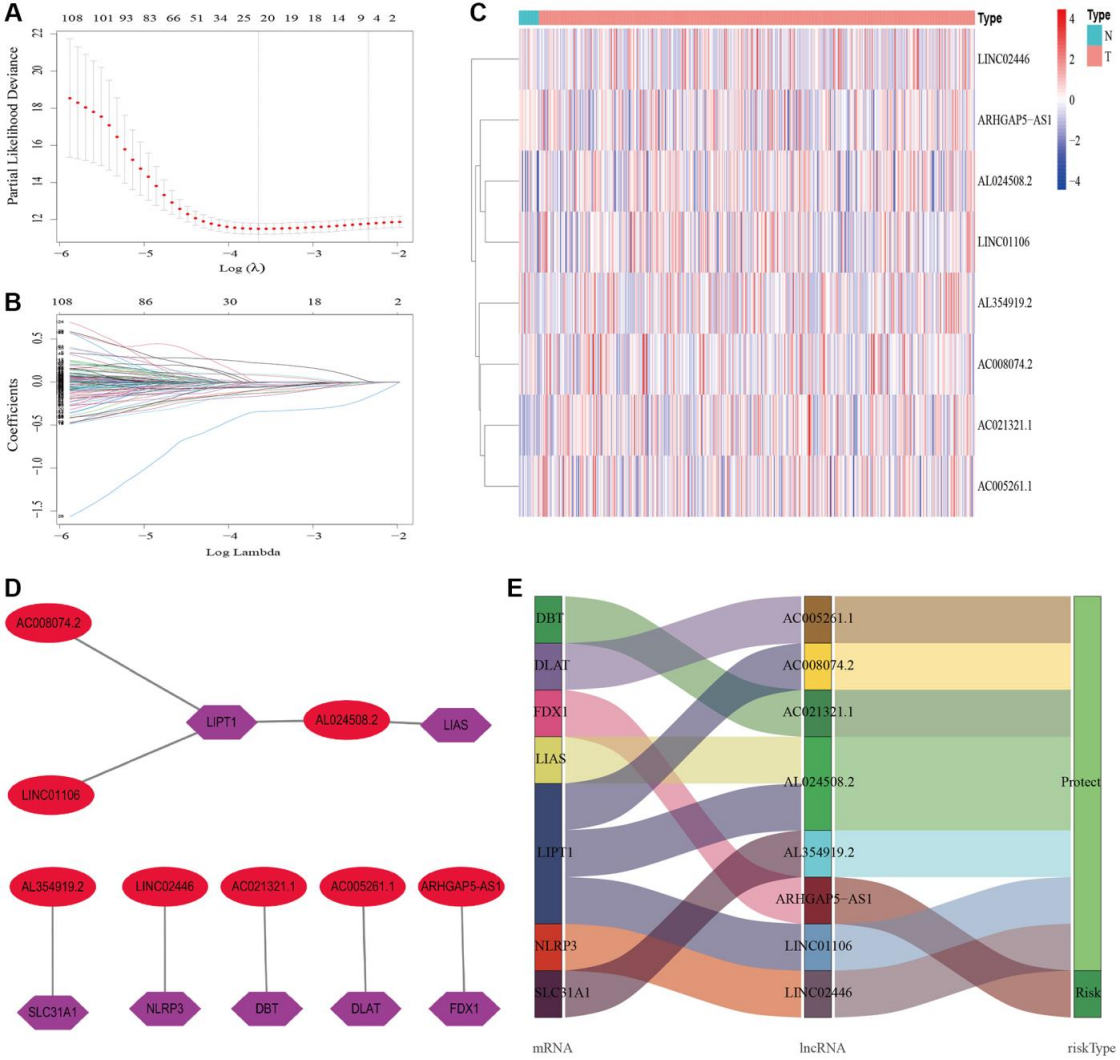
**Construction of the cuproptosis-related lncRNAs predictive signature.** (**A**) LASSO coefficient profiles of the expression of 135 lncRNAs. (**B**) Selection of the penalty parameter (λ) in the LASSO model via 20-fold cross-validation. (**C**) The expression levels of eight cuproptosis-related lncRNAs in tumor and normal tissues. (**D**) The co-expression network of prognostic cuproptosis-related lncRNAs. (**E**) Sankey diagram of prognostic cuproptosis-based lncRNAs. Abbreviations: lncRNAs: long noncoding RNAs; N: normal; T: tumor.

**Figure 3:**
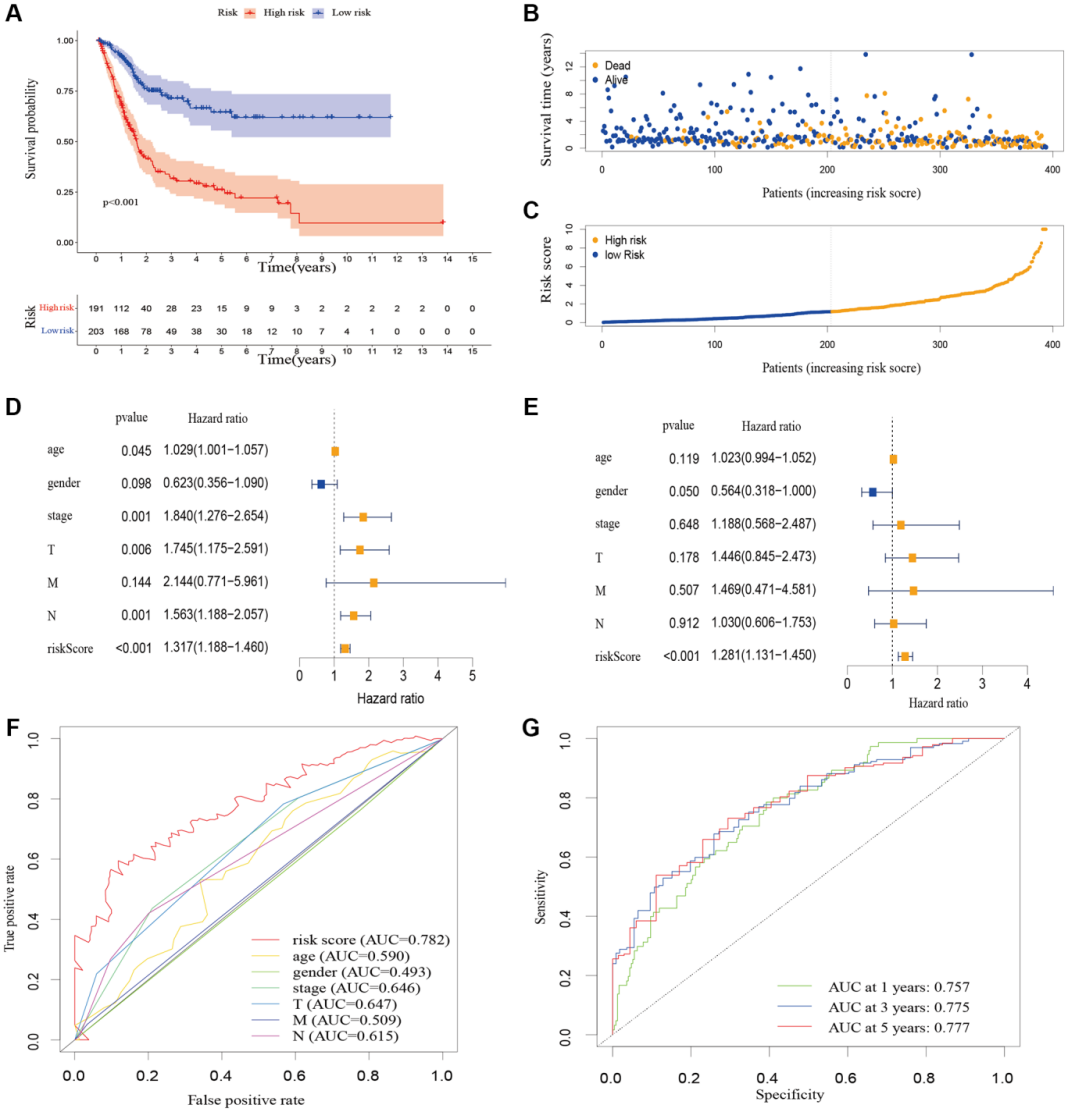
**The correlation between the predictive signature and the prognosis of BLCA patients.** (**A**) Kaplan-Meier analysis of the OS rate of BLCA patients in the high-risk and low-risk groups. (**B**) The distribution of the risk score among BLCA patients. (**C**) The number of dead and alive patients with different risk scores. Blue represents the number of survivors, and yellow represents the number of deaths. (**D**) Forest plot for univariate Cox regression analysis. (**E**) Forest plot for multivariate Cox regression analysis. (**F**) The ROC curve of the risk score and clinicopathological variables. (**G**) ROC curve and AUCs at 1-year, 3-years and 5-years survival for the predictive signature. Abbreviations: BLCA: Bladder urothelial carcinoma; OS: overall survival; ROC: receiver operating characteristic; AUC: area under the curve; T: tumor; N: lymph node; M: metastasis.

**Figure 4:**
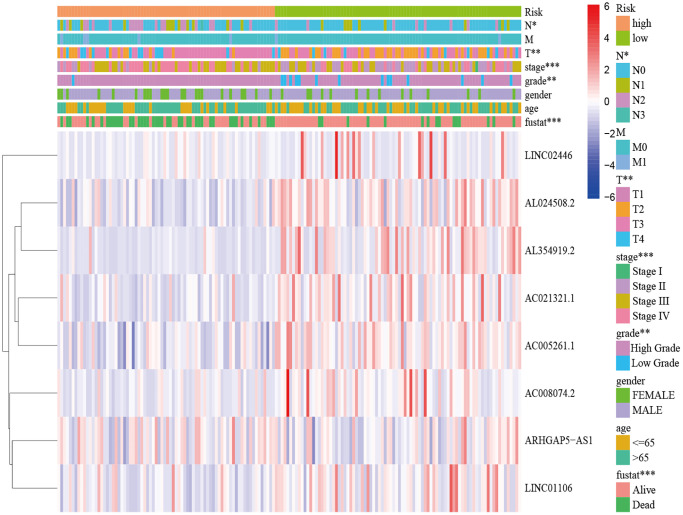
**Distribution heat map of five prognostic cuproptosis-related lncRNAs and clinicopathological variables in the high-risk and low-risk groups.** Abbreviations: lncRNAs: long noncoding RNAs; T: tumor; N: lymph node; M: metastasis.

A total of 548 cuproptosis-related lncRNAs were identified (Supplementary Table 1). Univariate Cox regression analysis revealed that 135 lncRNAs were associated with the prognosis of BLCA patients (Supplementary Figure 1). LASSO Cox regression model screened 20 lncRNAs (Figure 2A, 2B, Supplementary Table 2). 20 lncRNAs were subjected to multivariate Cox regression analysis to create a predictive signature, which identified a final set of 8 lncRNAs (AC005261.1, AC008074.2, AC021321.1, AL024508.2, AL354919.2, ARHGAP5-AS1, LINC01106, LINC02446). The expression heatmap of 8 cuproptosis-related lncRNAs in BLCA patients was shown in Figure 2C. We further visualized lncRNAs using Cytoscape software and the R package “ggalluvial”. The co-expression network contained 9 pairs of lncRNA-mRNAs (Figure 2D). AC005261.1 had co-expressive relationship with the DLAT; AC008074.2 had co-expressive relationship with LIPT1; AC021321.1 had co-expressive relationship with DBT; AL024508.2 had co-expressive relationship with LIAS and LIPT1; AL354919.2 had co-expressive relationship with SLC31A1; ARHGAP5-AS1 had co-expressive relationship with FDX1; LINC01106 had co-expressive relationship with LIPT1; LINC02446 had co-expressive relationship with NLRP3. AC005261.1, AC008074.2, AC021321.1, AL024508.2, AL354919.2, LINC01106 and LINC02446 were protective factors, while ARHGAP5-AS was risk factor (Figure 2E). The risk score was calculated as follows: Risk score = 0.372 × expression level of ARHGAP5-AS1 − 0.500 × expression level of AC005261.1 − 0.620 × expression level of AC008074.2 − 1.204 × expression level of AC021321.1 − 0.415 × expression level of AL024508.2 − 0.592 × expression level of AL354919.2 − 0.613 × expression level of LINC01106 − 0.738 × expression level of LINC02446. After evaluating the risk score of every BLCA patient using the aforementioned formula, the patients were stratified into high-risk group and low-risk group according to the median of the risk score. Compared with the low-risk group, Figure 3A showed that the high-risk group experienced considerably reduced OS time (*p* < 0.001). The risk scores of the high-risk and low-risk groups are shown in Figure 3B. As the risk score increased, univariate and multivariate Cox regression analyses were conducted on the available variables to ascertain their association with a higher number of patient fatalities (Figure 3C). Univariate Cox regression analysis indicated that age, stage, T stage, *N* stage and risk score showed a significant association with the OS (Figure 3D). Additionally, multivariate Cox regression analysis demonstrated that, according to Figure 3E, risk score was the independent predictor of OS. The risk score’s AUC was 0.782, which was the best among the available variables in predicting the prognosis of BLCA patients (Figure 3F). According to time-ROC analysis, the predictive signature has a great predictive performance, with AUCs of 0.757, 0.775 and 0.777 at 1-, 3-, and 5 years, respectively (Figure 3G). Furthermore, after analyzing the differences in clinicopathological variables between the high-risk and low-risk groups, we found that there were differences in stage (*p* < 0.001), T stage (*p* < 0.05), *N* stage (*p* < 0.05), grade (*P* < 0.01), and fustat (*p* < 0.001) between the high-risk and low-risk groups (Figure 4).

### Design of a nomogram

**Figure 5:**
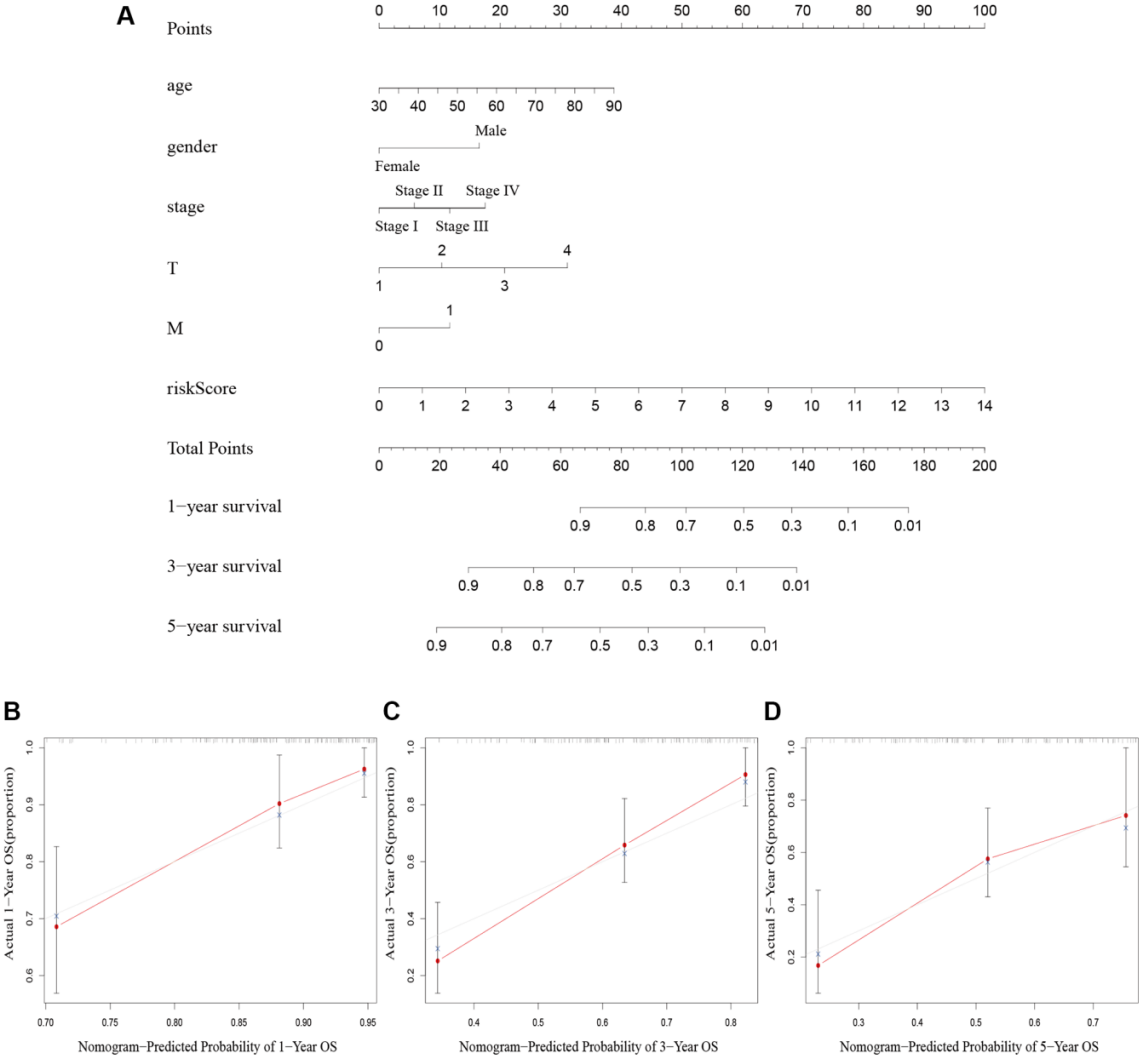
**Nomogram creation and validation.** (**A**) A nomogram combining clinicopathological variables and risk score predicts 1-year, 3-year, and 5-year OS rate of BLCA patients. (**B**–**D**) The calibration curves for the OS nomogram model in BLCA at 1-year, 3-year, and 5-year. Abbreviations: OS: overall survival; BLCA: Bladder urothelial carcinoma; N: lymph node; M: metastasis.

We created a nomogram including clinicopathological factors and risk score to simplify the clinical use of the predictive signature. The nomogram showed that it could help us predict the 1-, 3-, and 5-year survival rates of BLCA (Figure 5A). The calibration curves displayed a strong correlation between the predicted and actual 1-, 3-, and 5-year overall survival rates, as illustrated in Figure 5B–5D.

### Relationship between the predictive signature and the prognosis of BLCA patients among the clinicopathological variables

**Figure 6:**
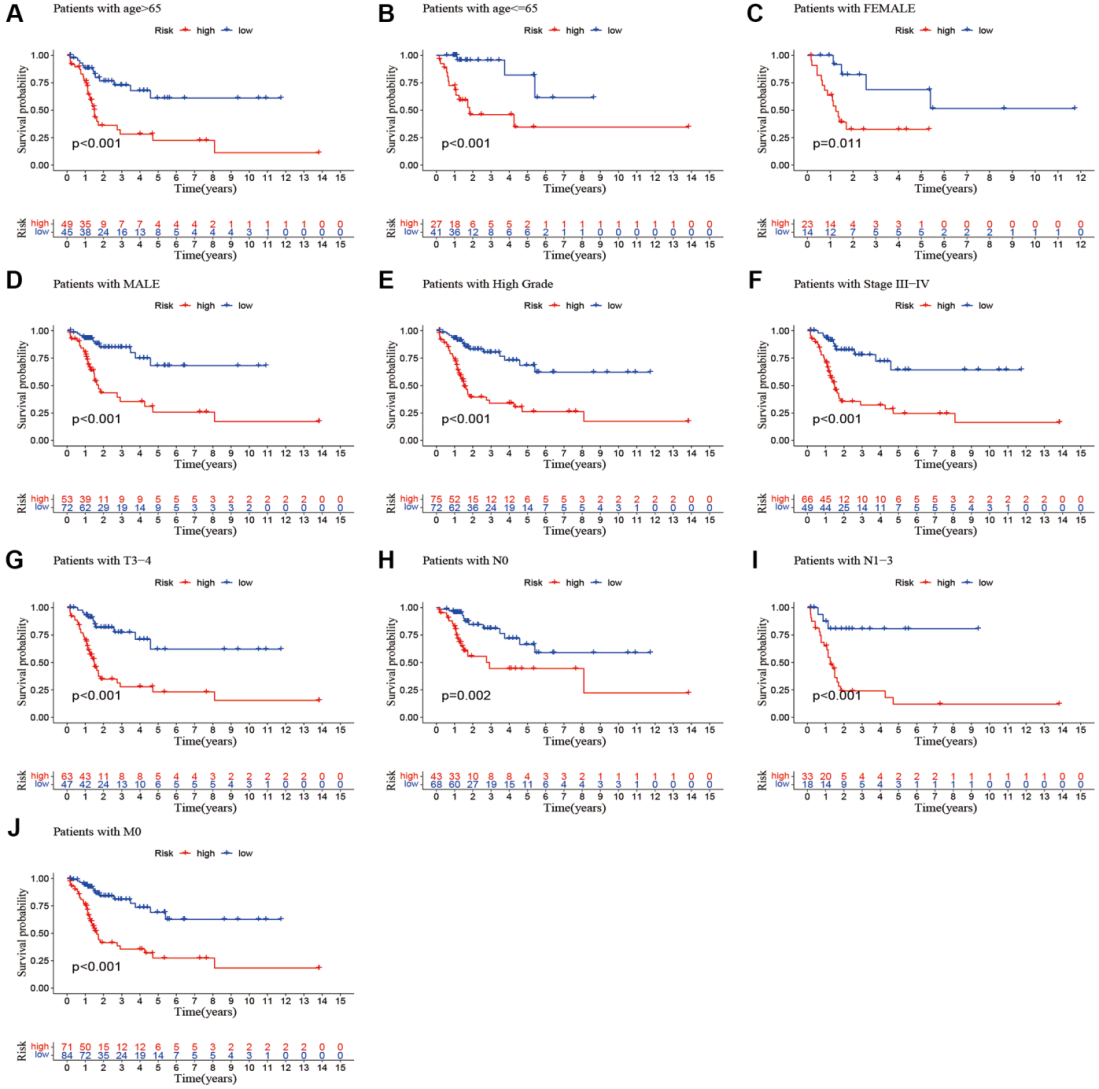
**Kaplan-Meier survival curves of high-risk and low-risk groups among patients sorted according to different clinicopathological variables.** (**A**, **B**) Age; (**C**, **D**) Gender; (**E**) High Grade; (**F**) Stage III–IV; (**G**) T3–4 stage; (**H**, **I**) *N* Stage; (**J**) M0 stage. Abbreviations: T: tumor; N: lymph node; M: metastasis.

We performed a subgroup analysis of the different age, gender, grade, stage and Tumor Node Metastasis (TNM) stage to study the relationship between the predictive signature and the prognosis of BLCA patients. We found that for the age, gender, high grade, stage III–IV, T3–4 stage, *N* stage and M0 stage, the OS of BLCA patients in the high-risk group was shorter than that in the low-risk group (Figure 6).

### Internal validation of the cuproptosis-related lncRNA predictive signature

**Figure 7:**
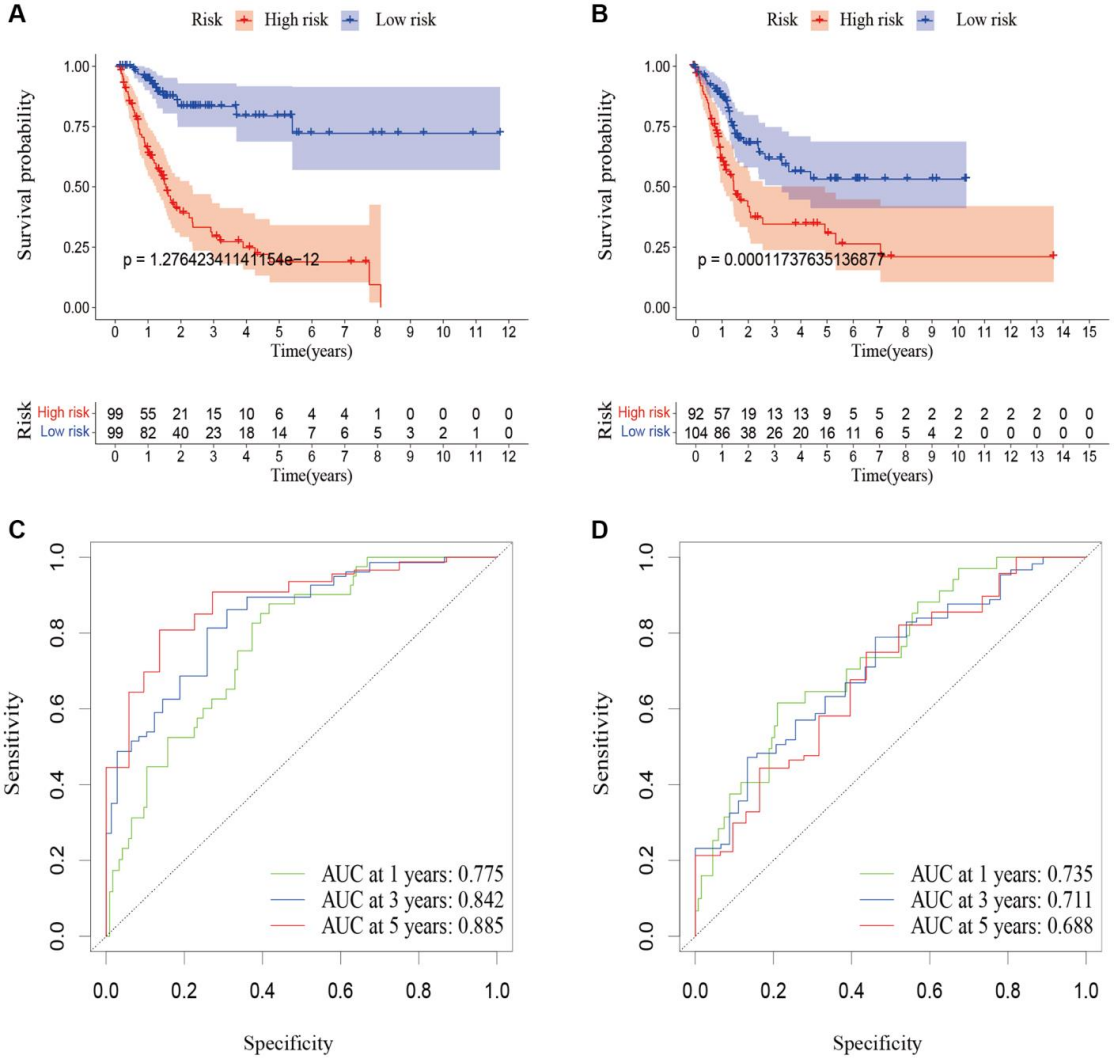
**Validation of the predictive signature for OS based on the entire TCGA dataset.** (**A**) Kaplan-Meier survival curve in the training cohort. (**B**) Kaplan-Meier survival curve in the testing cohort. (**C**) ROC curve and AUCs at 1-year, 3-years and 5-years survival in the training cohort. (**D**) ROC curve and AUCs at 1-year, 3-years and 5-years survival in the testing cohort. Abbreviations: OS: overall survival; TCGA: The Cancer Genome Atlas. ROC: receiver operating characteristic; AUC: area under the curve.

To verify the accuracy of the predictive signature for OS based on the entire TCGA cohort, we randomly divided the 394 BLCA patients into two cohorts, training cohort and testing cohort (Table 1). In the training cohort, the OS rate of patients in the high-risk group was lower than that of the low-risk group (Figure 7A, *p* = 1.276e-12).

According to Figure 7C, the AUC for 1-year survival was 0.775, 3-year survival was 0.842, and 5-year survival was 0.885. The prognosis of patients in the high-risk group in the testing cohort was also poorer than that of the low-risk group (Figure 7B, *p* = 0.0001). According to Figure 7D, the AUC for 1-year survival was 0.735, for 3-year survival it was 0.71, and for 5-year survival it was 0.688. These outcomes were in line with those of the entire TCGA cohort, proving that the predictive signature can serve as a reliable predictor of a patient’s prognosis for BLCA.

### Functional enrichment analysis between different risk patients

**Figure 8:**
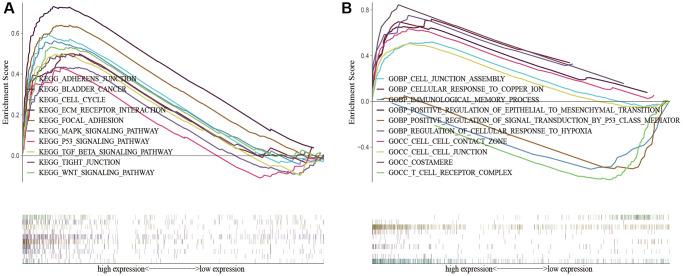
**KEGG and GO enrichment analysis in the predictive signature between high-risk and low-risk groups.** (**A**) KEGG enrichment analysis; (**B**) GO enrichment analysis. Abbreviations: KEGG: Kyoto Encyclopedia of Genes and Genomes; GO: Gene Ontology.

To demonstrate the biological functions and pathways linked with risk score among patients with different risk levels, we conducted a functional enrichment analysis. By performing GSEA on the high-risk and low-risk groups, we were able to detect possible differences between them. As anticipated, we observed significant enrichment of immune-related biological processes and tumor-related signaling pathways in the high-risk group compared to the low-risk group. These pathways included cell cycle, ECM receptor interaction, tight junction, WNT signaling pathway, MAPK signaling pathway, P53 signaling pathway, TGF-beta signaling pathway, cell-cell junction, cellular response to copper ion, immunological memory process, T cell receptor complex, regulation of cellular response to hypoxia, etc. (Figure 8).

### Immune infiltration level analysis

**Figure 9:**
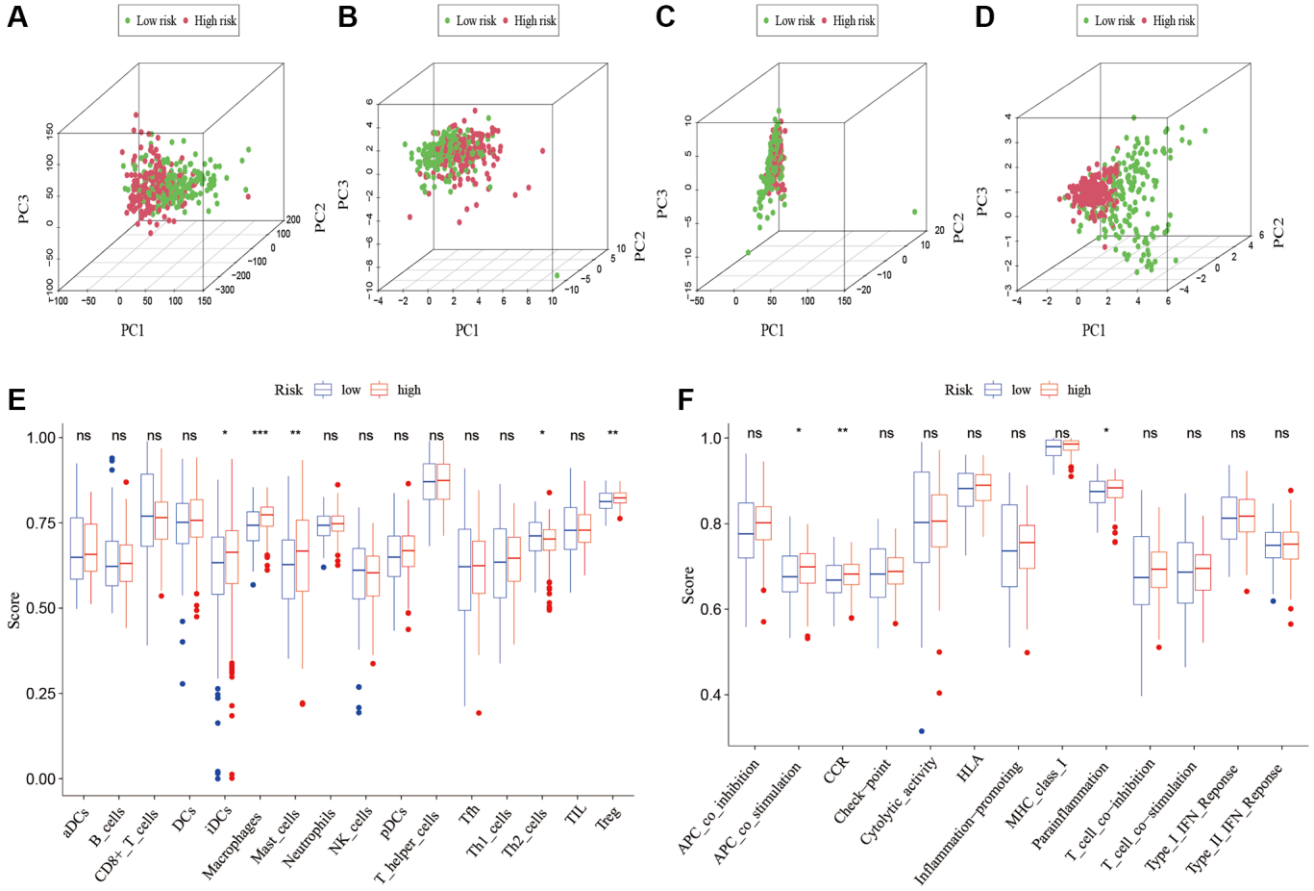
**Patients with high-risk and low risk scores have different immune statuses.** PCA maps show the distribution of patients based on the (**A**) whole genome; (**B**) cuproptosis-related gene sets; (**C**) cuproptosis-related lncRNAs; and (**D**) the predictive signature. Results for ssGSEA scores immune cells scores (**E**) and immune functions scores (**F**) between high and low risk groups in boxplots. Abbreviations: PCA: Principal component analysis; lncRNAs: long noncoding RNAs; ssGSEA: single-sample gene set enrichment analysis. aDCs: activated dendritic cells; iDCs: immature dendritic cells; NK: natural killer; pDCs: plasmacytoid dendritic cells; Tfh: T follicular helper; Th1: T helper type 1; Th2: T helper type 2; TIL: tumor-infiltrating lymphocyte; Treg: T regulatory cell; APC: antigen-presenting cell; CCR: chemokine receptor; HLA: human leukocyte antigen; MHC: major histocompatibility complex; IFN: interferon.

**Figure 10:**
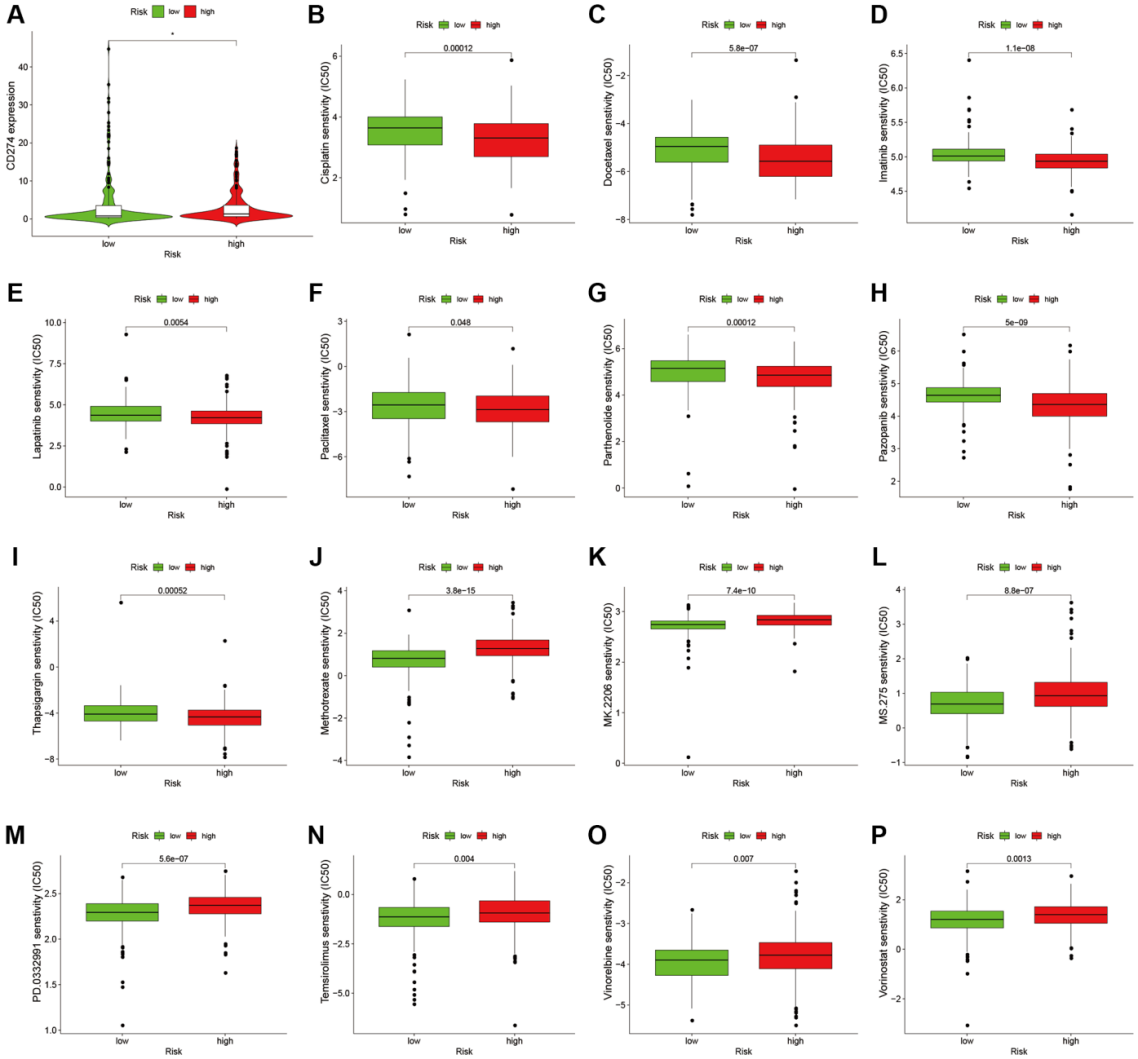
**Comparison of treatment drugs sensitivity between high-risk and low-risk groups.** (**A**) PD-L1 expression in high and low risk groups. (**B**) IC50 of cisplatin in high and low risk groups. (**C**) IC50 of docetaxel in high and low risk groups. (**D**) IC50 of imatinib in high and low risk groups. (**E**) IC50 of lapatinib in high and low risk groups. (**F**) IC50 of paclitaxel in high and low risk groups. (**G**) IC50 of parthenolide in high and low risk groups. (**H**) IC50 of pazopanib in high and low risk groups. (**I**) IC50 of thapsigargin in high and low risk groups. (**J**) IC50 of methotrexate in high and low risk groups. (**K**) IC50 of MK.2206 in high and low risk groups. (**L**) IC50 of MS.275 in high and low risk groups. (**M**) IC50 of PD.0332991 in high and low risk groups. (**N**) IC50 of temsirolimus in high and low risk groups. (**O**) IC50 of vinorelbine in high and low risk groups. (**P**) IC50 of vorinostat in high and low risk groups. Abbreviation: IC50: half-maximal inhibitory concentration.

To analyze the level of immune infiltration, we utilized Principal component analysis (PCA) maps that enabled us to visualize patients’ distribution based on their whole genome, cuproptosis-related gene sets, cuproptosis-related lncRNAs, and predictive signature. The results showed that the predictive signature was the best for patients (Figure 9A–9D). Moreover, to investigate the association between risk scores and immune cells and pathways, we calculated the enrichment scores of ssGSEA concerning diverse immune cell subsets and their related pathways. The analysis confirmed the differences of immature dendritic cells (iDCs), macrophages, mast cells, T helper type 2 (Th2) cells, T regulatory cells (Tregs), chemokine receptor (CCR) and parainflammation between two risk groups (Figure 9E, 9F). Given the importance of checkpoint immunotherapy, furthermore observed to differ between two risk groups. When compared to the low-risk group, the high-risk group’s PD−L1 expression was much higher. Results indicated that anti-PD-1/L1 immunotherapy may be effective in high-risk individuals (Figure 10A). Along with immunotherapy, we also analyzed the effect of common drugs on the efficacy of BLCA. The results showed a lower IC50 value of cisplatin, docetaxel, imatinib, lapatinib, paclitaxel, parthenolide, pazopanib and thapsigargin in the high-risk group (Figure 10B–10I), but a higher IC50 value of methotrexate, MK.2206, MS.275, PD.0332991, temsirolimus, vinorelbine and vorinostat in the high-risk group (Figure 10J–10P), which aids in investigating specialized therapy options for BLCA patients in the high-risk and low-risk categories.

### The correlations between risk scores/8 cuproptosis-related lncRNAs and clinicopathological variables

**Figure 11:**
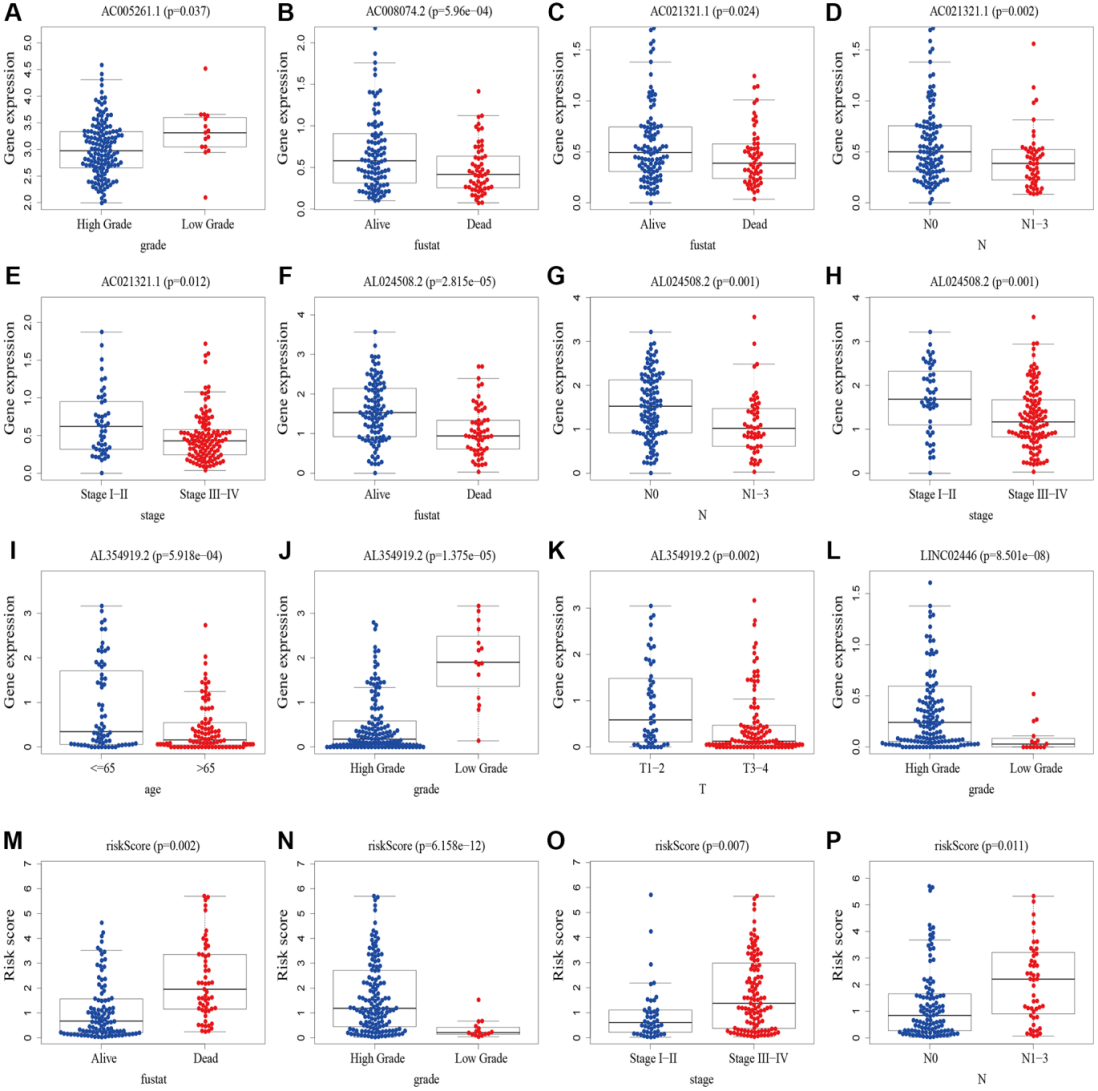
**The correlations between risk scores/8 cuproptosis-related lncRNAs and clinicopathological variables.** (**A**) Correlation between AC005261.1 expression level and grade. (**B**) Correlation between AC008074.2 expression fustat. (**C**–**E**) Correlation between AC021321.1 expression level and fustat, *N* stage and stage. (**F**–**H**) Correlation between AL024508.2 expression level and fustat, *N* stage and stage. (**I**–**K**) Correlation between AL354919.2 and age, grade and T stage. (**L**) Correlation between LINC02446 expression level and M stage. (**M**–**P**) Correlation between risk scores expression level and fustat, grade, stage and *N* stage. Abbreviations: lncRNAs: long noncoding RNAs; T: tumor; N: lymph node; M: metastasis.

We analyzed the correlation between clinicopathological variables and risk scores/8 cuproptosis-related lncRNAs using gene expression and corresponding clinical data from the TCGA database. Results showed that AC005261.1 was correlated with grade (Figure 11A); AC008074.2 was associated with fustat (Figure 11B); AC021321.1 was correlated with fustat, grade, stage, T stage and *N* stage (Figure 11C–11E, Supplementary Figure 2A, 2B); AL024508.2 was associated with fustat, grade, stage, T stage and *N* stage (Figure 11F–11H, Supplementary Figure 2C, 2D); AL354919.2 was correlated with age, fustat, grade, stage, and TNM stage (Figure 11I–11K, Supplementary Figure 2E–2H); LINC02446 was associated with grade and M stage (Figure 11L, Supplementary Figure 2I); risk scores were correlated with fustat, grade, stage, T stage and *N* stage (Figure 11M–11P, Supplementary Figure 2J).

### Validation of the expression of the cuproptosis-related lncRNAs in indicated cell lines

**Figure 12:**
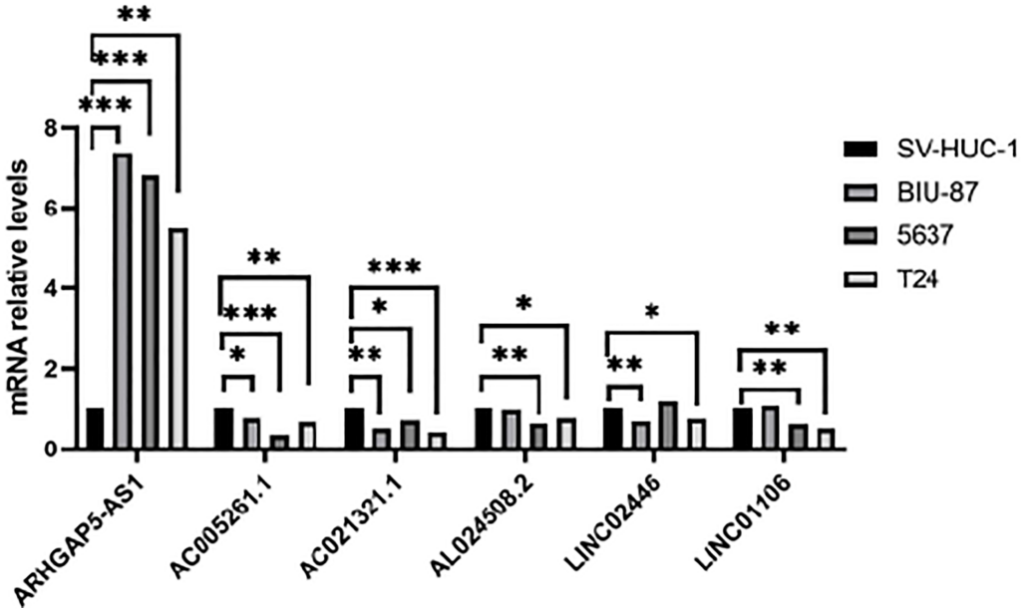
**Validation of results through quantitative PCR.** Relative mRNA expression of the cuproptosis-related lncRNAs in four cell lines (SV-HUC-1, 5637, BIU-87 and T24).

We chose three BLCA cell lines to examine the levels of their mRNA expression in order to further evaluate the expression of the cuproptosis-related lncRNAs. The control group was normal bladder cell line SV-HUC1. The results were shown in Figure 12. LncRNA AC005261.1, AC021321.1, AL024508.2, LINC02446 and LINC01106 were lowly expressed in tumor cells, while ARHGAP5-AS1 showed the opposite trend.

## DISCUSSION

Copper at appropriate concentrations is involved in many metabolic processes, but excess copper ions can be toxic at elevated concentrations [25]. Studies showed that copper homeostasis was closely related to tumorigenesis and development, and the cytotoxicity caused by its imbalance could regulate cancer cell growth and proliferation. Cuproptosis [26], a new form of cell death, was reported firstly by Tsvetkov et al. which could be regulated and controlled by copper ions [13]. The use of copper ion to kill cancer cells is a potential new treatment for cancers. Although there have been some studies on cuproptosis, it has not been reported to predict the prognosis of BLCA patients by constructing cuproptosis-related lncRNA predictive signature.

In the present study, we first obtained the cuproptosis-related genes (CRGs) and the related lncRNAs of these genes. We conducted univariate Cox regression analysis to investigate the association between cuproptosis-related lncRNAs and the prognosis of patients with bladder cancer (BLCA). After performing LASSO Cox regression model screening, we obtained 20 lncRNAs. Then, we identified 8 cuproptosis-related lncRNAs (AC005261.1, AC008074.2, AC021321.1, AL024508.2, AL354919.2, ARHGAP5-AS1, LINC01106, LINC02446) through multivariate Cox regression analysis for inclusion to create a predictive signature. Previous studies have shown that AC008074.2, AC021321.1, AL354919.2, ARHGAP5-AS1, LINC01106 and LINC02446 could predict the prognosis and be used as the prognostic markers of BLCA [27–33]. LINC01116 could regulate ELK3 and HOXD8 to promote bladder cancer cells proliferation, migration, and invasion [34]. Liyuan Zhu et al. discovered that chemoresistant gastric cancer cells have an upregulated level of the lncRNA ARHGAP5-AS1. While doing so, in individuals with gastric cancer, a high expression of ARHGAP5-AS1 was linked to a bad prognosis [35]. According to Xiaotong Zhang et al., bladder cancer cells proliferation and metastasis were inhibited by the lncRNA LINC02446 [36]. Seven mRNAs (DBT, DLAT, FDX1, LIAS, LIPT1, NLRP3 and SLC31A1) significantly co-expressed with above lncRNAs. Increased glycolytic metabolism and PM2.5-activated DLAT overexpression have been shown to accelerate the development of non-small cell lung cancer, suggesting that DLAT may be the therapeutic marker of this type of cancer [37]. According to Zeyu Zhanget et al., FDX1 can affect prognosis and is closely associated to lung adenocarcinoma’s glucose metabolism, fatty acid oxidation, and amino acid metabolism [38]. A pan-cancer analysis showed that various patients’ excellent prognoses were linked to high LIAS expression. In addition, furthermore, LIAS expression had the ability to forecast the effectiveness of immunotherapy in cancer patients [39]. The risk of BLCA, tumor size, and lymph node metastasis are all related to the NLRP3 polymorphism [40]. The low level of NLRP3 in renal cancer suggests that NLRP3 may play a tumor suppressor role in RCC [41]. Moreover, when NLRP3 was overexpressed, Yi-Fan Tan et al. found that it inhibited cell proliferation and EMT progression in renal cancer cells [42]. In previous study, SLC31A1-dependent copper level was associated with the degree of malignancy of pancreatic cancer [43].

After being divided into high-risk and low-risk groups, the OS of patients in the high-risk group was shorter compared to those in the low-risk group. The ROC curve indicated that the predictive signature had good predictive ability. The predictive signature was more credible than clinicopathological variables in predicting the prognosis of BLCA patients. Therefore, risk score was an independent predictor of OS. Internal validation showed that the predictive signature had good prediction ability as well. Based on the GSEA enrichment results we found that the cuproptosis-associated lncRNA predictive signature we constructed in this study was not only associated with the tumor-related signaling pathways, but also the immune-related biological processes.

Potential treatment targets in various risk groups of bladder cancer patients can be discovered by examining the infiltrating impact of immune cells on tumor microenvironment and immune checkpoint genes. Our results of ssGSEA showed that iDCs, macrophages, mast cells and Tregs had higher infiltration scores in the high-risk group. Tumor-associated macrophages, especially M2 macrophages, are actively involved in tumor progression in glioma patients [44]. High stromal tumor mast cell infiltration is an independent adverse prognostic factor in MIBC patients. Patients with lower stromal tumor mast cell levels may benefit more from adjuvant chemotherapy [45]. Study showed that Tregs potentially regulated BLCA invasiveness [46]. However, in terms of immune checkpoints, most of the genes involved were highly expressed in high-risk groups. Our research also shows that high-risk groups are probably sensitive to anti-PD-1/L1 immunotherapy and cisplatin, docetaxel, imatinib, lapatinib, paclitaxel, parthenolide, pazopanib and thapsigargin, but are resistant to methotrexate, MK.2206, MS.275, PD.0332991, temsirolimus, vinorelbine and vorinostat. Above results suggest that the combination of immunotherapy and other drug treatment can benefit the high-risk groups and can provide a personalized treatment for BLCA patients.

Our study does have certain drawbacks, though. First of all, our data were from retrospective studies in the TCGA database and were not prospective. We still need data from other databases or our own patients for external validation. Cellular and animal models are needed to validate these results. Second, the mechanism of cuproptosis-related lncRNAs in BLCA needs to be further verified by experiments.

In conclusion, we successfully constructed a strong predictive signature of 8 cuproptosis-related lncRNAs. This signature can independently predict the prognosis of BLCA patients. This signature also offers a promising strategy for potential anticancer immunotherapy.

## MATERIALS AND METHODS

### Publicly accessible data processing and collecting

The clinical information for TCGA-BLCA patients as well as the fragments per kilobase of transcript per million mapped reads (FPKM)-standardized RNA-seq data were downloaded from TCGA website (https://portal.gdc.cancer.gov/) [47] on August 2022. A total of 394 BLCA patients with lncRNA expression values and survival times and 19 normal samples were involved in our study. The log2 (FPKM + 1) transformation was used to normalize the transcriptome data. The gene annotations were obtained from the GENCODE project (Homo sapiens GRCh38) [48]. The CRGs (FDX1, SLC31A1, LIAS, LIPT1, LIPT2, DLD, DLAT, PDHA1, PDHB, MTF1, GLS, CDKN2A, DBT, GCSH, DLST, NFE2L2, NLRP3, ATP7B, ATP7A) were obtained from previous studies [11–13, 49, 50]. We subsequently explored for their expression and prognostic value in BLCA.

### Construction of the cuproptosis-related lncRNA predictive signature

To develop a predictive signature for cuproptosis-related lncRNAs, we initially utilized the “limma” package to correlate lncRNAs with CRGs through Pearson correlation analysis, setting criteria of correlation coefficient|R²| > 0.3 and *P*-value of < 0.001. Next, we employed univariate Cox regression analysis to screen for lncRNAs that were related to patient prognosis within the group of cuproptosis-related lncRNAs. Then we used the R packages “glmnet”, “survminer”, “caret”, and “survival” to construct the cuproptosis-related lncRNA predictive signature through LASSO Cox regression model. The formula utilized in this analysis is Risk score = ∑iCoefficient (lncRNAi) × Expression (lncRNAi).

### PPI network analysis

The interactions between the prognostic cuproptosis-related lncRNAs and CRGs were determined through the protein-protein interaction (PPI) network analysis using the STRING database [51] and visualized by Cytoscape software [52].

### Creation of nomogram

We created a nomogram utilizing the R package “rms” to predict the 1-, 3-, and 5-year survival of BLCA patients by combining the risk score with clinicopathological characteristics. We used calibration curves to test coefficient prediction efficacy.

### Functional enrichment analysis of the lncRNA predictive signature by GSEA

According to the median risk score for GSEA (version 4.2.3) enrichment analysis [53], patients were split into high- and low-risk groups for functional enrichment analysis of the cuproptosis-related lncRNA prediction signature. The potential functions of the CRGs were investigated using Kyoto Encyclopedia of Genes and Genomes (KEGG) and Gene Ontology (GO) enrichment analysis. FDR of 0.25 and a typical *P*-value of 0.05 in the GSEA analysis indicated a meaningful difference. Single sample gene set enrichment analysis (ssGSEA) was used to quantify immune cells and pathways by the R package “GSVA”.

### The role of the predictive signature in predicting the clinical treatment response

We compared the half-maximum inhibitory concentration (IC50) values of common drugs for the clinical treatment between high-risk and low-risk groups by the R package “pRRophetic”.

### Cell culture

The SV-HUC-1, T24, BIU-87 and 5637 cell lines used in this study were purchased from the Chinese Academy of Science in Shanghai. SV-HUC-1 cell line were cultured F12 medium (Procell), T24, BIU-87 and 5637 cell lines were cultured RPMI-1640 medium (Procell) and supplemented with 10% FBS (Biological Industries) at 37°C in a 5% CO_2_ humidified atmosphere.

### RNA isolation and quantitative RT-PCR

RNA was isolated from cells by using TRIZOL reagent (TaKaRa). cDNA was then synthesized using HiScript^®^ III All-in-one RT SuperMix Perfect for qPCR (Vazyme). qRT-PCR for mRNA was performed on the StepOne Plus Real-Time PCR system (Applied Biosystems). The relative mRNA level was calculated as a 2^−ΔΔCt^ value and normalized against β-actin. PCR primer sequences are listed in Supplementary Table 3.

### Statistical analysis

The R language (version 4.2.0) was used to perform statistical analysis. The Kaplan-Meier method was performed along with log-rank test to analyze the survival of patients. The “survivalROC” package was used to draw the ROC curves for presenting the prediction ability. PCA was used to investigate the distribution of patients with different risk scores. Univariate Cox regression analysis was used to analyze the relationship between cuproptosis-related lncRNAs and OS. Additionally, multivariate Cox regression analyses were performed to recognize autonomous predictors of OS. *P* < 0.05 was considered statistically significant difference.

### Availability of data and materials

The datasets used and/or analyzed during the current study are available from the corresponding author on reasonable request.

## Supplementary Materials

Supplementary Figures

Supplementary Table 1

Supplementary Tables 2 and 3
