# Supplementary material for: A novel cuproptosis-related lncRNAs signature predicts prognosis in bladder cancer
Source: Aging (Albany NY). 2023 Jul 9;15(13):6445–66. doi: 10.18632/aging.204861 (PMC10373974; doi:10.18632/aging.204861)
Supplement: Supplementary Figures [file aging-15-204861-s001.pdf]

SUPPLEMENTARY FIGURES

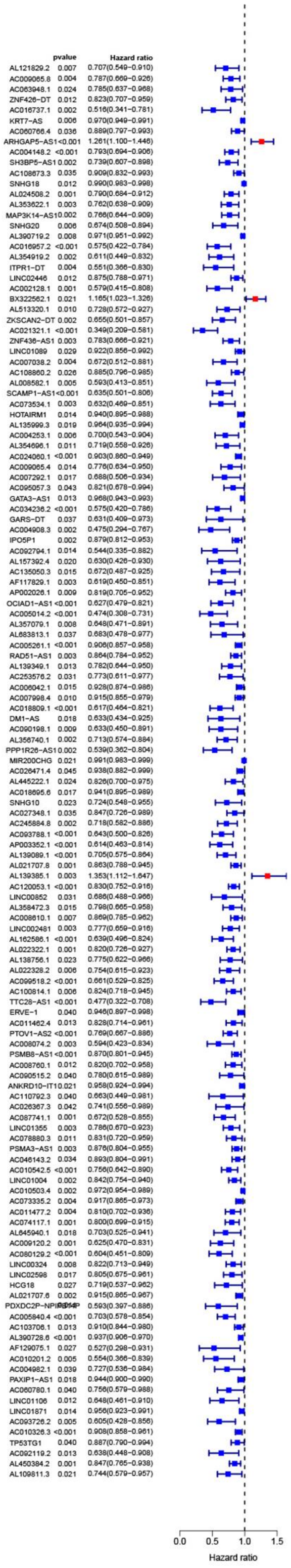

Supplementary Figure 1. A total of 135 lncRNAs analyzed by Univariate Cox regression analysis.

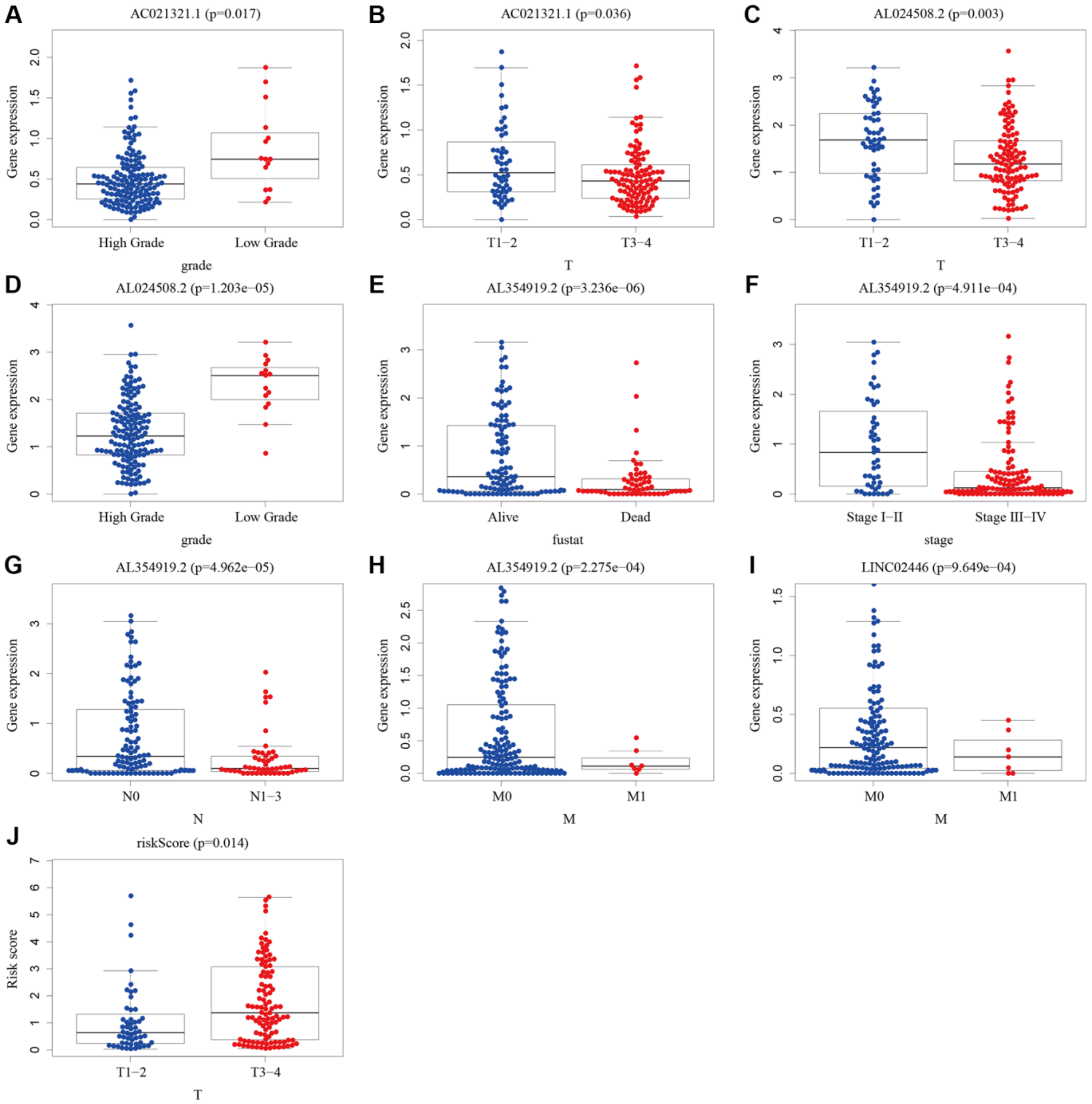

Supplementary Figure 2. The correlations between risk scores/8 cuproptosis-related lncRNAs and clinicopathological variables. (**A**, **B**) Correlation between AC021321.1 expression level and grade and T stage. (**C**, **D**) Correlation between AL024508.2 expression level and grade and T stage. (**E**–**H**) Correlation between AL354919.2 expression level and fustat, stage *N* stage and M stage. (**I**) Correlation between LINC02446 and M stage. (**J**) Correlation between risk scores expression level and T stage. Abbreviations: lncRNAs: long noncoding RNAs; T: tumor; N: lymph node; M: metastasis.
