# Supplementary material for: A novel cuproptosis-related lncRNAs signature predicts prognosis in bladder cancer
Source: Aging (Albany NY). 2023 Jul 9;15(13):6445–66. doi: 10.18632/aging.204861 (PMC10373974; doi:10.18632/aging.204861)
Supplement: Supplementary Tables 2 and 3 [file aging-15-204861-s003.pdf]

SUPPLEMENTARY TABLES

Supplementary Table 2. A total of 20 lncRNAs screened by LASSO Cox regression model.

ID
AL121829.2
ARHGAP5-AS1
SNHG18
AL024508.2
AL354919.2
LINC02446
BX322562.1
AC021321.1
AL135999.3
OCIAD1-AS1
AC005261.1
AL356740.1
AP003352.1
AL139385.1
AL162586.1
AC099518.2
AC008074.2
PSMB8-AS1
LINC02598
LINC01106

Supplementary Table 3. Oligonucleotide sequences used in this study.

Primes and probes		Sequences
AC005261.1	Forward	5′-CAGCACAGGTCTCAGCCTCATT-3′
	Reverse	5′-GCCAGGGAAGTTCACTCAAGCAT-3′
AC021321.1	Forward	5′-CGCACACAGGTTCCCTAATGTTTACT-3′
	Reverse	5′-GCACACAGGTTCCCTAATGTTTACT-3′
AL024508.2	Forward	5′-AGCAGCACTGGACACACAAGAGA-3′
	Reverse	5′-CATCATCACAGACCACAGCAGAAGT-3′
ARHGAP5-AS1	Forward	5′-TGTTCACGCCACTACCAGCCTAA-3′
	Reverse	5′-TCACAGGACCTCAGTTTCTTCAATGG-3′
LINC02446	Forward	5′-CCAGTAACAGGCAAGAAGAGAATAGAGG-3′
	Reverse	5′-ACATCGTAGGAGGTGCTGTCAGAATA-3′
LINC01106	Forward	5′-CAGGAGGTCTGGATCTGTGATGAGA-3′
	Reverse	5′-TCTTGAGCCCACTTTCCCGATCT-3′
β-actin	Forward	5′-CATGTACGTTGCTATCCAGGC-3′
	Reverse	5′-CTCCTTAATGTCACGCACGAT-3′
